# Influence of Thermal Treatment Conditions on the Properties of Dental Silicate Cements

**DOI:** 10.3390/molecules21020233

**Published:** 2016-02-18

**Authors:** Georgeta Voicu, Alexandru Mihai Popa, Alina Ioana Badanoiu, Florin Iordache

**Affiliations:** 1 Department of Science and Engineering of Oxide Materials and Nanomaterials, Faculty of Applied Chemistry and Material Science, Politehnica University of Bucharest, 1-7 Gh. Polizu Street, Bucharest RO-011061, Romania; georgeta.voicu@upb.ro (G.V.); alexbpa_md@yahoo.com (A.M.P.); 2 Department of Fetal and Adult Stem Cell Therapy, Nicolae Simionescu Institute of Cellular Biology and Pathology of Romanian Academy, 8 B.P. Hasdeu Street, Bucharest RO-050568, Romania; loriniordache84@yahoo.com

**Keywords:** mineral trioxide cement, composition, thermal treatment, hydration and hardening processes, properties, setting time, biocompatibility

## Abstract

In this study the sol-gel process was used to synthesize a precursor mixture for the preparation of silicate cement, also called mineral trioxide aggregate (MTA) cement. This mixture was thermally treated under two different conditions (1400 °C/2 h and 1450 °C/3 h) followed by rapid cooling in air. The resulted material (clinker) was ground for one hour in a laboratory planetary mill (v = 150 rot/min), in order to obtain the MTA cements. The setting time and mechanical properties, *in vitro* induction of apatite formation by soaking in simulated body fluid (SBF) and cytocompatibility of the MTA cements were assessed in this study. The hardening processes, nature of the reaction products and the microstructural characteristics were also investigated. The anhydrous and hydrated cements were characterized by different techniques e.g., X-ray diffraction (XRD), scanning electron microscopy (SEM), infrared spectroscopy (FT-IR) and thermal analysis (DTA-DTG-TG). The setting time of the MTA cement obtained by thermal treatment at 1400 °C/2 h (MTA1) was 55 min and 15 min for the MTA cement obtained at 1450 °C/3 h (MTA2). The compressive strength values were 18.5 MPa (MTA1) and 22.9 MPa (MTA2). Both MTA cements showed good bioactivity (assessed by an *in vitro* test), good cytocompatibility and stimulatory effect on the proliferation of cells.

## 1. Introduction

Dental silicate cements, also known as mineral trioxide aggregate (MTA) cements, are currently used in endodontic procedures for root perforation repairs and root-canal sealing [1,2,3,4,5,6,7,8,9,10,11]. The mineral phases present in MTA cement are similar with those present in Portland cement, e.g., calcium silicates (3CaO∙SiO_2_ and 2CaO∙SiO_2_) and tricalcium aluminate (3CaO∙Al_2_O_3_). For practical reasons MTA cement paste should be radioopaque therefore bismuth oxide can also be added to its formulations [1,2].

Our research group has reported the synthesis of mineralogical compounds specific for MTA cements e.g., 2CaO∙SiO_2_ [12] and 3CaO∙Al_2_O_3_ [13] as well as MTA cements [14] using a non-conventional method—the sol-gel route. Compared with the solid state reaction route, the usual synthesis method for this type of materials, the sol-gel route presents some advantages like high purity of the resulted products and lower thermal treatment temperatures. Moreover, the MTA cements obtained by the sol-gel method have good biocompatibility and no recordable cytotoxicity [14].

The setting time of MTA silicate cements, obtained by our group using this method, is still too long [14,15], especially if dental applications are the aim; therefore, in this paper we present new MTA cement formulation, with a higher amount of 3CaO∙Al_2_O_3_, compound with high reactivity *vs.* water. The influence of thermal treatment parameters (temperature and plateau) on the main properties of MTA cement, before and after hardening, is also presented in this paper.

## 2. Results and Discussion

**Figure 1:**
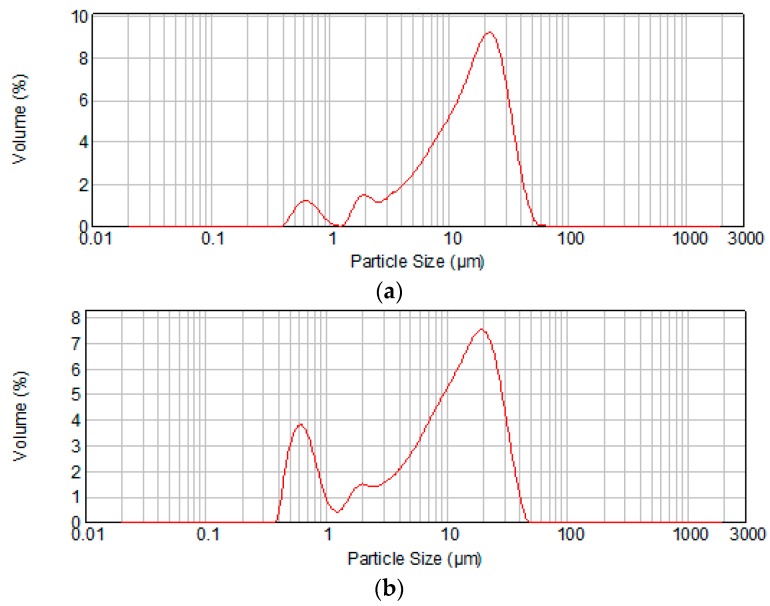
Grain size distribution of MTA cements obtained at: (**a**) 1400 °C/2 h (MTA1); (**b**) 1450 °C/3 h (MTA2).

The powers obtained by the grinding of MTA clinkers for 15 min in a planetary mill, were analyzed by laser granulometry. The main granulometric characteristics of the two MTA cements are presented in Table 1 and the grains size distributions curves are presented in Figure 1.

The data presented in Table 1 confirms the higher fineness of MTA2 powder as compared with MTA1. The amount of grains with particles sizes below 1 μm is higher for the MTA cement obtained at higher temperature *i.e.*, 1450 °C/3 h (MTA2) as compared with the one obtained at 1400 °C/2 h (MTA1)—Figure 1.

**Figure 2:**
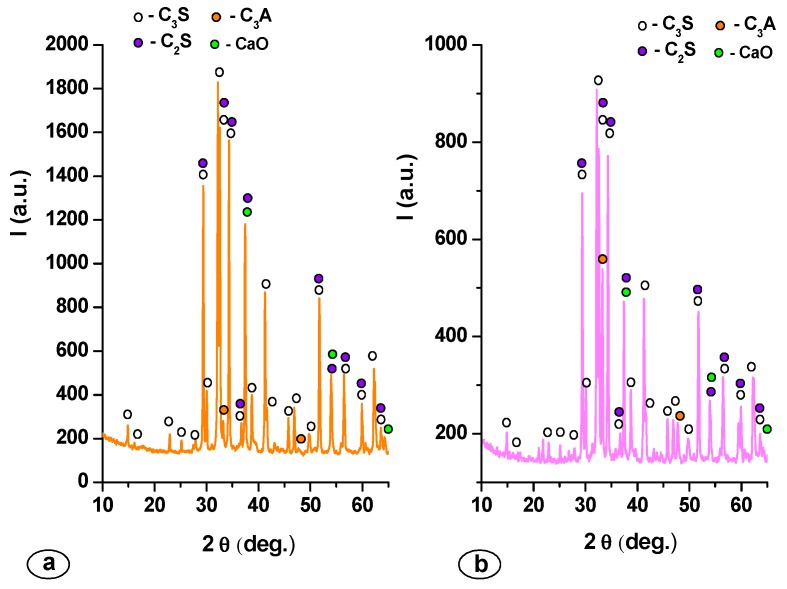
XRD patterns of MTA cements: (**a**) MTA1 and (**b**) MTA2.

Information regarding the crystalline compounds formed during the thermal treatment in MTA cements were obtained by XRD (Figure 2).

The main mineralogical crystalline compounds assessed by this technique in both MTA1 and MTA2 cements are: tricalcium silicate—3CaO∙SiO_2_ (C_3_S) (JCPDS 42-0551), dicalcium silicate—2CaO∙SiO_2_ (C_2_S) (JCPDS 76-0799), tricalcium aluminate—3CaO∙Al_2_O_3_ (C_3_A) (JCPDS 38-1429) and free lime (JCPDS 37-1497). The results obtained by Rietveld refinement of XRD patterns are presented in Table 2.

The first thermal treatment was performed at 1400 °C with a 2 h plateau based on previous results reported in other papers [14,15,16]. This thermal treatment ensured the formation of calcium silicates and tricalcium aluminate, but the amount of free lime was high (4.3%), which implies that a higher amount of mineralogical compounds can be obtained if the thermal treatment temperature and plateau increase. Therefore, the second thermal treatment was performed at 1450 °C for 3 h. As expected, the increase of thermal treatment temperature (from 1400 °C to 1450 °C) and plateau (from 2 h at 3 h) determines a certain increase of the amount of C_3_S and C_3_A, compounds with a high reactivity *vs.* water [17,18]. Also, the amount of free lime decreases from 4.3% (MTA1) to 1.1% (MTA2); for this type of cements, free lime amount can also play an important role due to its antibacterial properties [2,7,19,20,21]. The setting time and compressive strength values of MTA cements are presented in Table 3.

The important decrease of the MTA2 setting time (as compared with MTA1) can be due to several factors e.g.,:

(i) the higher amount of C_3_S and C_3_A formed in MTA2 ( as compared with MTA1)—see Table 2. Both tricalcium silicate and tricalcium aluminates are mineralogical compounds with high reactivity *vs.* water [17] and actively contributes to decrease of the setting time;
(ii) the higher amount of small cement grains in MTA2 as compared with MTA1 (see Table 1 and Figure 1) contributes also to the important decrease of the setting time noticed for the cement thermally treated at higher temperature.

The values of the compressive strengths, assessed after 7 and 28 days of hardening, are comparable for the two studied MTA cements (Table 3). Both setting time and compressive strengths have values similar with those reported in the literature for silicate dental cements [9,22].

The compressive strengths values of hardened MTA cements depend both on the nature and amount of hydrates formed during hardening process as well as the microstructure of hardened pastes.

**Figure 3:**
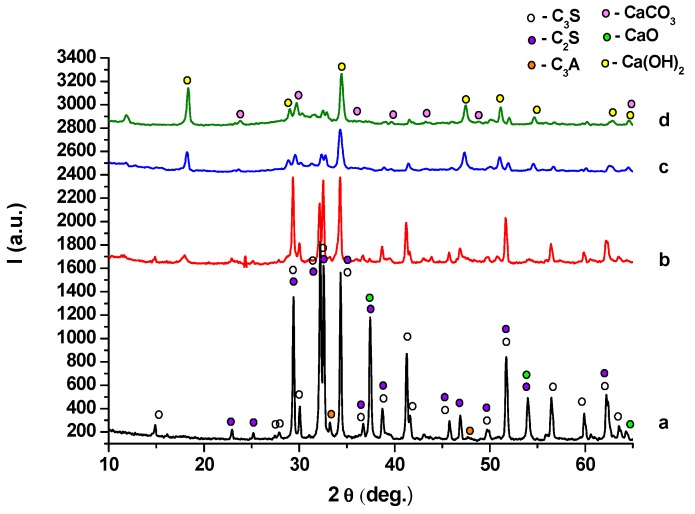
XRD patterns of MTA1: (**a**) anhydrous; (**b**) hydrated for 1 day; (**c**) hydrated for 7 days; (**d**) hydrated for 28 days.

**Figure 4:**
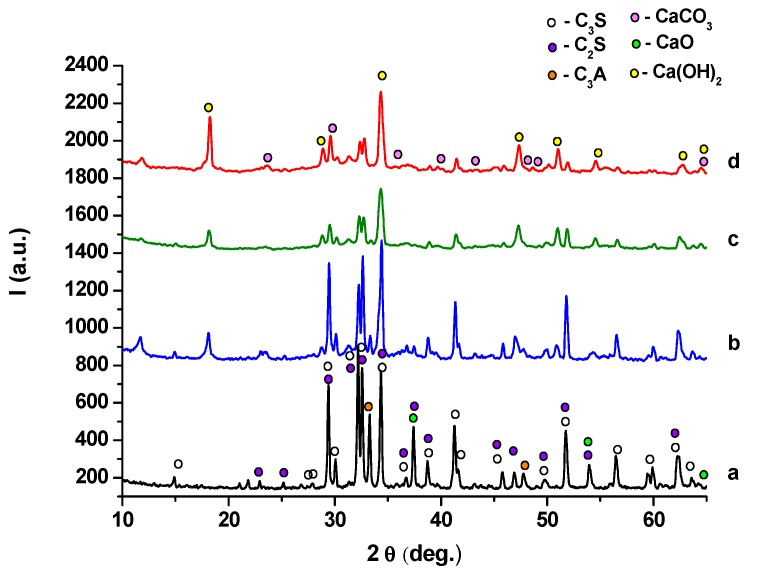
XRD patterns of MTA2: (**a**) anhydrous; (**b**) hydrated for 1 day; (**c**) hydrated for 7 days; (**d**) hydrated for 28 days.

XRD patterns of anhydrous MTA cements and cement pastes hydrated for 1, 7 and 28 days, presented in Figure 3b–d and Figure 4b–d, provide information regarding the composition of these materials.

For the hydrated cement pastes (Figure 3 and Figure 4), a decrease of the intensity of peaks specific for anhydrous compounds (C_3_S, C_2_S and C_3_A) due to their consumption in hydration processes it can be noticed. The only crystalline hydrate formed during MTA cement hydration is portlandite—Ca(OH)_2_; the intensity of its specific peaks increases with the increase of the hydration time, a clear indication of its formation in a higher amount. On the XRD patterns of both MTA cements pastes are also present the peaks specific for calcium carbonate—this compound is most probably formed due to the carbonation of portlandite with atmospheric CO_2_ [18].

**Figure 5:**
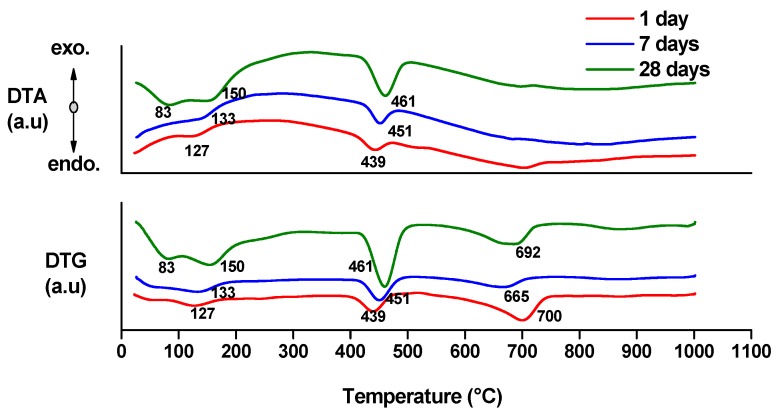
DTA and DTG curves of MTA1 paste hydrated for 1, 7 and 28 days.

**Figure 6:**
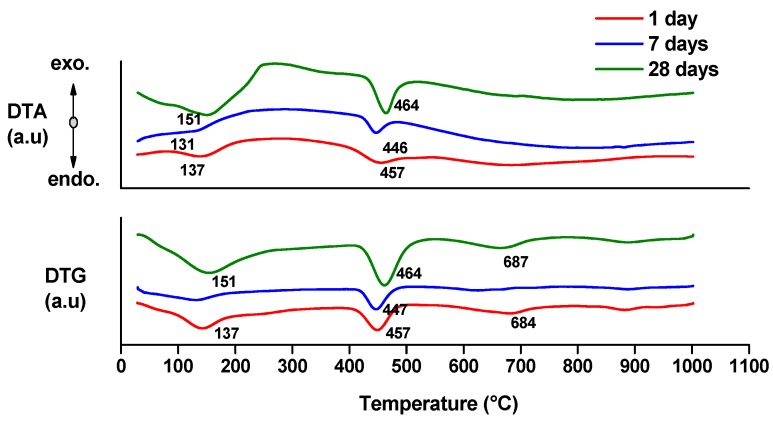
DTA and DTG curves of MTA2 paste hydrated for 1, 7 and 28 days.

Thermal analysis can provide quantitative information about the hydrates (gel or crystalline compounds) [17,18]. The endo- effects present on the DTA curves (Figure 5 and Figure 6) associated with the weight loss assessed on the DTG and TG curves can be attributed to the following processes [17,18]:

- endo-effects recorded up to 100 °C are due to the loss of moisture;
- endo-effects recorded between 100 and 220 °C are due to the dehydration of gel like calcium silicates hydrates and calcium aluminate hydrates formed by cement hydration;
- endo-effects recorded between 400 and 500 °C are determined by the dehydration of portlandite;
- the endo-effects recorded between 600 and 850 °C are attributed to the decarbonation of CaCO_3_ with different crystallization degrees; as previously presented, this compound is most probably formed due to the carbonation of portlandite with atmospheric CO_2_.

**Figure 7:**
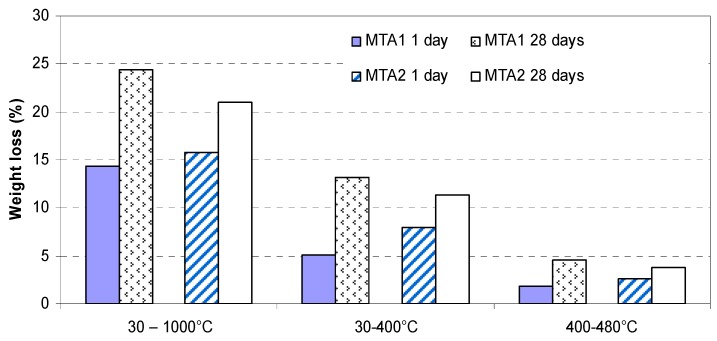
Weight losses recorded on TG curves of MTA cement pastes hydrated for 1 day and 28 days.

The weight losses recorded on TG curves were processed and are presented in Figure 7.

As it can be seen from Figure 7, the total weight loss (recorded between 30 and 1000 °C) increases from 1 day to 28 days for both MTA cements, due to the progress of hydration processes. The weight loss corresponding to the 30–400 °C interval provides information about the amount of calcium silicates hydrates (C-S-H) and calcium aluminate hydrates (C-A-H) formed in the hydrated pastes; one can notice the higher amount of C-S-H and C-A-H formed after 1 day of hydration in MTA2 paste as compared with MTA1 paste—this can be correlated with the higher rate of hydration in the first system, confirmed also by the lower values of the setting time (Table 3). The higher amount of portlandite (assessed by the weight loss between 400 and 480 °C) formed after 1 day of hydration in MTA2 paste, as compared with MTA1 paste, confirms also the higher hydration rate of MTA2 cement, at early ages.

**Figure 8:**
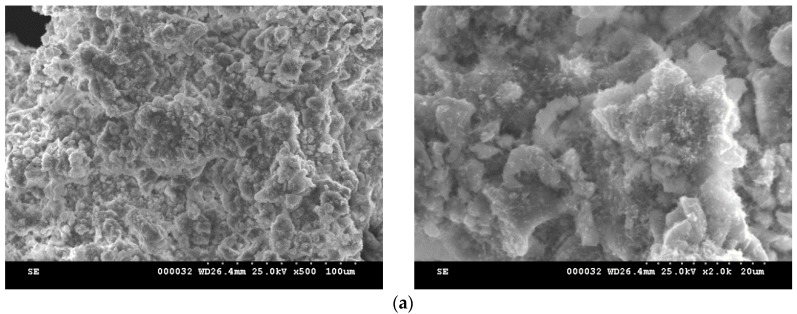

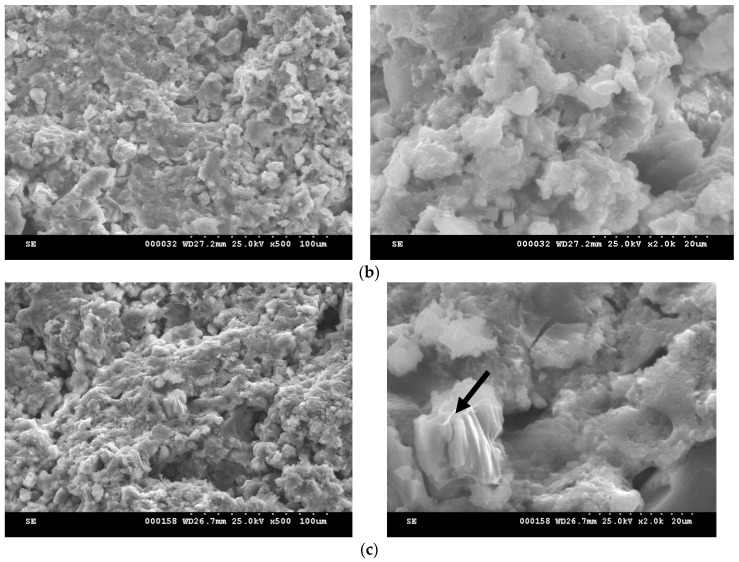
SEM images of MTA1 pastes hydrated for: (**a**)—1 day; (**b**)—7 days; (**c**)—28 days.

**Figure 9:**
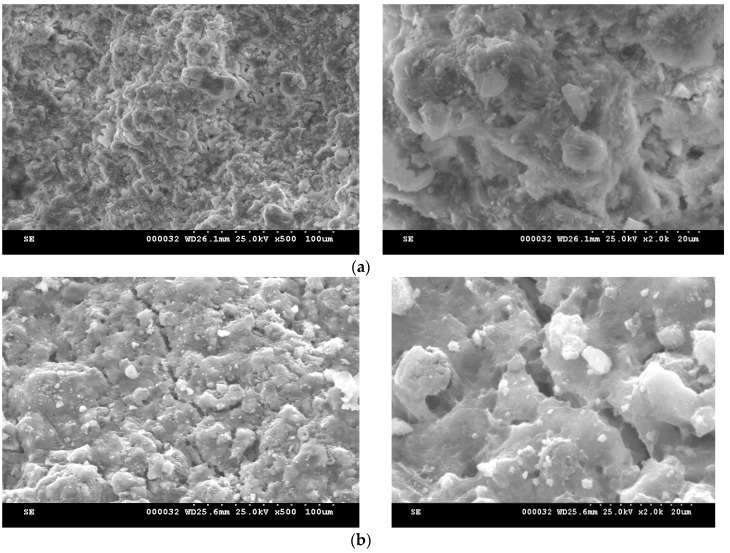

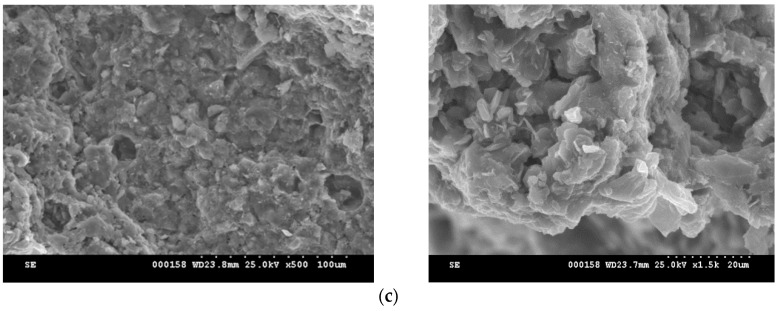
SEM images of MTA2 pastes hydrated for: (**a**)—1 day; (**b**)—7 days; (**c**)—28 days.

The microstructure of MTA cement pastes, hydrated different periods of time, are presented in Figure 8 and Figure 9. The main hydrate phases assessed by XRD and DTA and TG, e.g., the calcium silicates hydrates and portlandite, can be also assessed in the SEM images; calcium silicates hydrates have specific morphologies *i.e.*, needle-like and plate microcrystals for calcium silicate hydrates and spongy agglomeration specific for carbonate phases [17,23]. In the microstructure of MTA1 paste can be also assessed the hexagonal plate crystals specific for of portlandite (see arrow in Figure 8c). The microstructure of MTA2 seems to be also more compact as compared with the one assess for MTA1; this is in good correlation with the slightly higher values of compressive strength developed by MTA2 pastes after 7 and 28 days of hydration as compared with MTA1.

*In vitro* bioactivity tests were conducted on MTA pastes immersed in SBF at 37 °C for 14 days.

**Figure 10:**
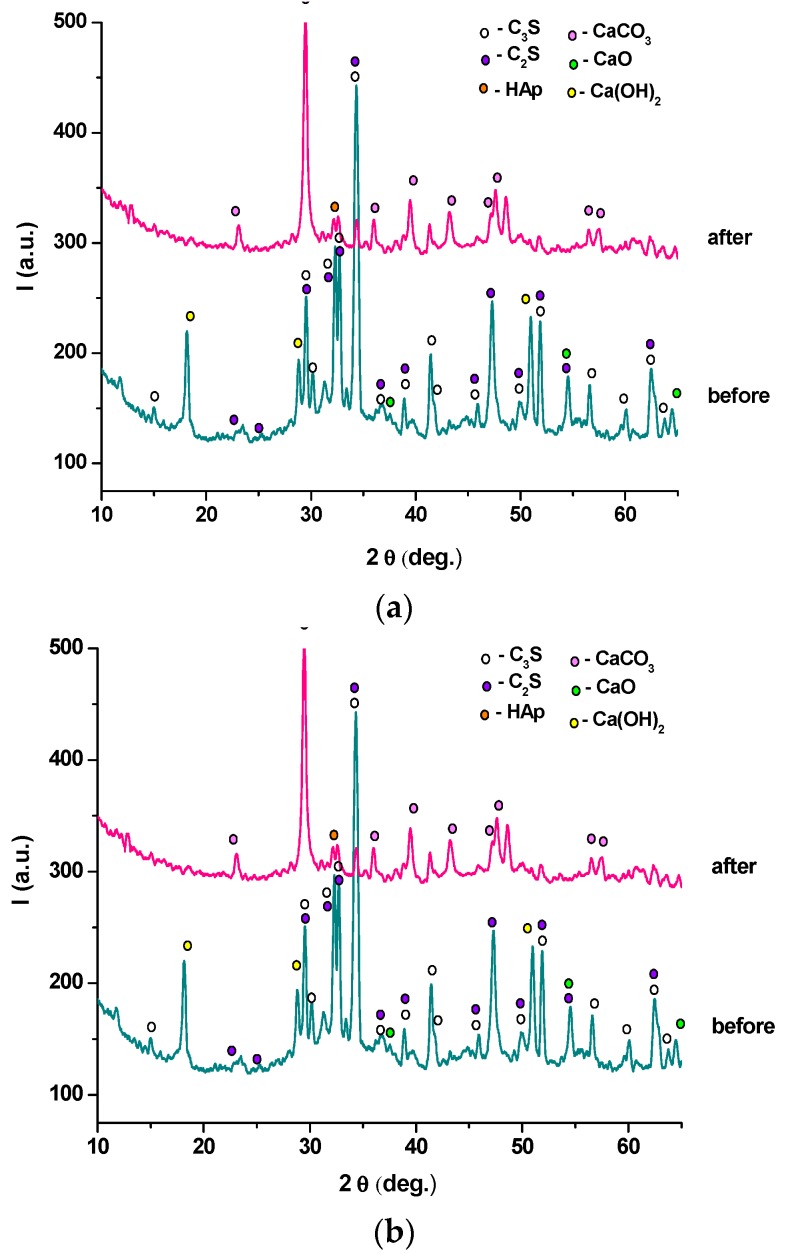
XRD patterns of hydrated MTA pastes before and after immersion in SBF for 14 days at 37 °C: (**a**) MTA1; (**b**) MTA2.

The XRD patterns of these pastes (Figure 10) show a decrease of the intensities of diffraction peaks specific for anhydrous compounds (C_3_S, C_2_S) and portlandite (Ca(OH)_2_) confirming an interaction of these phases with SBF; also, on the XRD spectra of MTA pastes immersed for 14 days in SBF, new peaks specific for hydroxyapatite (HAp) (JCPDS 84-1998) and calcium carbonate (JCPDS 03-0596) are found. The presence of HAp, compound with very good biocompatibiliy, is a clear indication of an adequate behavior of these materials if used as dental cements.

**Figure 11:**
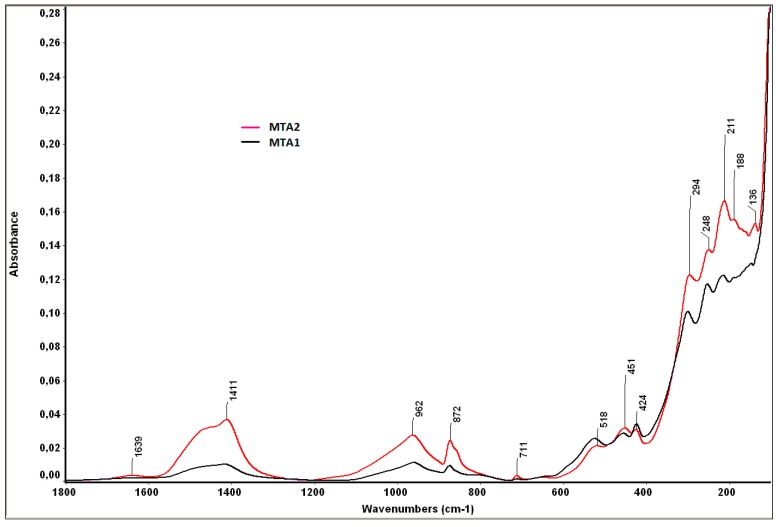
FT-IR spectra of MTA pastes hydrated for 7 days and the immersed SBF for 14 days.

The FT-IR analysis (Figure 11) of MTA pastes immersed for 14 days in SBF shows the following specific bands [24,25,26]:

- bands at 1424 cm^−1^ and 872 cm^−1^, specific for calcium carbonate, also assessed by XRD;
- bands at 962 cm^−1^ and 518 cm^−1^, specific for the phosphate group (PO_4_^3−^), from HAp;
- the band of 1411 cm^−1^ is specific for carbonated HAp formed by the substitution of PO_4_^3−^ with CO_3_^2−^;
- the band of 1639 cm^−1^ is specific for hydroxyl groups (moisture).

Also, one can assess on the FT-IR spectra specific bands for hydrated phases—calcium silicates hydrates (451, 962, 1411 and 1639 cm^−1^), calcium aluminate hydrate (424 cm^−1^), or for anhydrous compounds (e.g., dicalcium silicate at 518 cm^−1^); the bands between 400 and 150 cm^−1^ can be also attributed to the polymerized silicate tetrahedra, present in the silicate hydrate structure.

**Figure 12:**
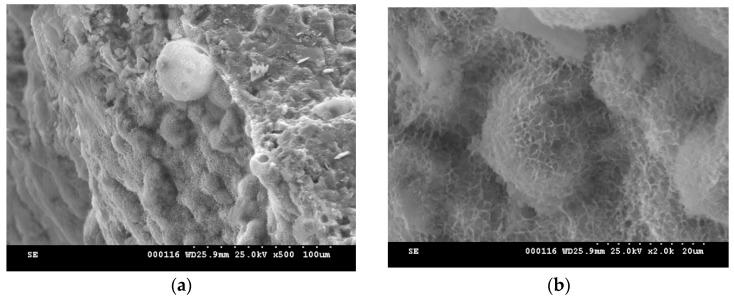

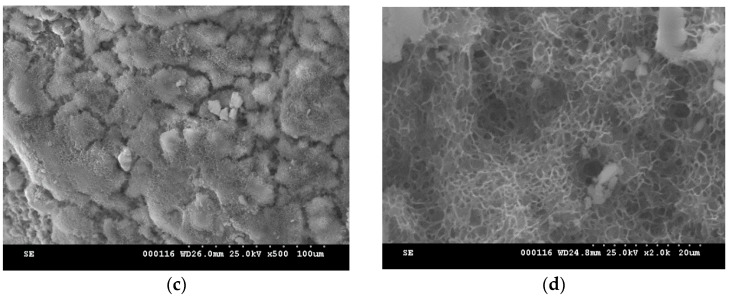
SEM micrographs of MTA pastes hydrated for 7 days and the immersed SBF for 14 days at 37 °C: (**a**), (**b**)—MTA1; (**c**), (**d**)—MTA2.

The SEM images of MTA pastes, immersed for 14 days in SBF (Figure 12), shows the presence of a thin friable layer formed by agglomerations of plate like crystals; this morphology is associated to HAp [27,28,29].

The cytotoxicity of MTA was verified by a MTT assay that is based on biochemical reactions that measure the metabolic activity of living cells. The MTT assay demonstrated that the human endothelial cells presented normal metabolism and growth in the presence of MTA.

**Figure 13:**
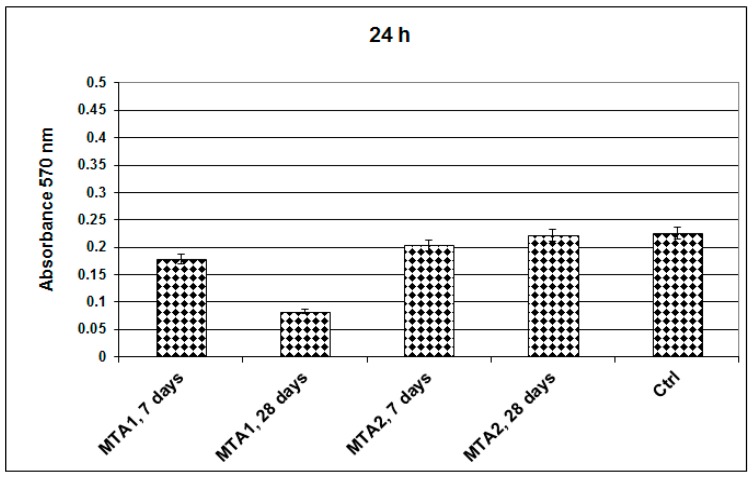

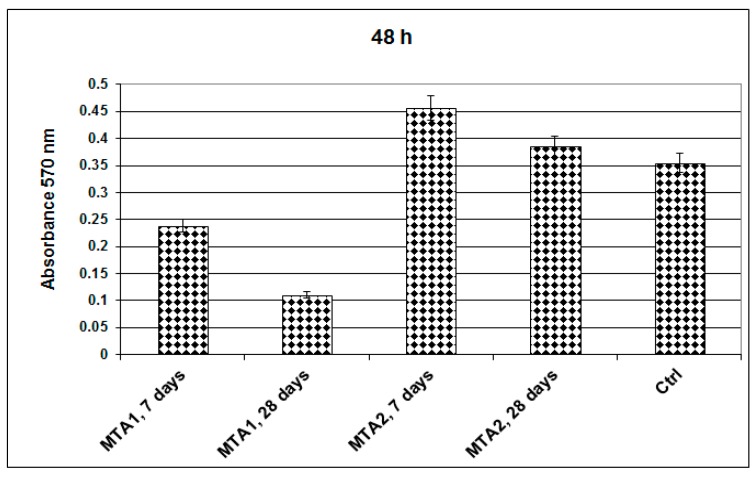
Endothelial cells proliferation profiles after growing on control (ctrl) and MTA for up to 48 h.

**Figure 14:**
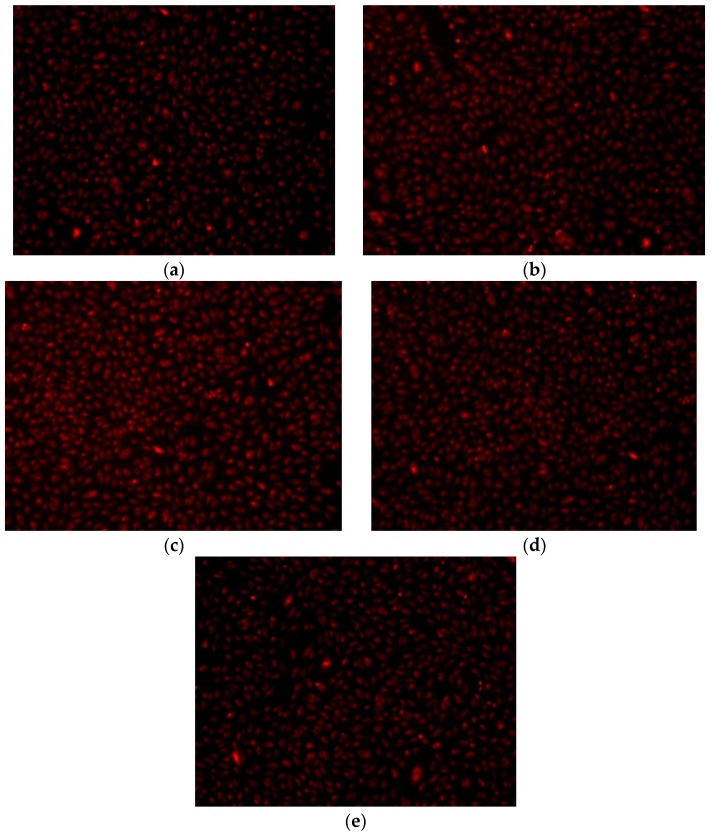
Fluorescence microscopic images of the endothelia cell monolayer in the presence of MTA (x20): (**a**) MTA1—7 days; (**b**) MTA1—28 days; (**c**) MTA2—7 days; (**d**) MTA2—28 days; (**e**) Ctrl.

The absorbance values measured at 570 nm showed a better proliferation of endothelial cells grown on MTA2 (1450 °C/3 h) compared to those grown on MTA1 (1400 °C/2 h) (Figure 13); this can be due to high basicity in the MTA1 system (with a high amount of free lime—Table 2). The fluorescent microscopy images confirm the biochemical viability test results showing that the endothelial cell viability is maintained after 24 h in the presence of MTA hydrated for 7 and 28 days. The endothelial cells retain normal morphology, were adherent and have a relatively uniform distribution on all investigated surfaces (Figure 14). The size and shape of MTA are important for the interaction with living cells. The average particle size measured by laser granulometry was 15.03 μm for MTA1 and 10.77 μm for MTA2, respectively. At these sizes the proliferation of endothelial cells was slightly decreased for MTA1—7 and 28 days. The MTA2 did not alter the cellular metabolism and moreover the proliferation was increased compared with control cells. The morphology was not modified, the endothelial cells retained a normal shape compared to control, adhered to culture plates, and had a relatively uniform distribution, being viable up to 7 days in the presence of MTA 1 and MTA2. The *in situ* synthesis of the MTA enables the cell to proliferate better compared with control cells, demonstrating the biocompatibility potential of these cements and confirming their potential application in dentistry. These results are consistent with those of other researchers that showed that different MTA cements have a good compatibility (80%–130% compared to the control group) with human osteoblastic cells [30,31].

## 3. Experimental Section

The oxide composition for MTA cement was: CaO—69.4%, SiO_2_—21.6%, Al_2_O_3_—5.8% and ZnO—3.2%. ZnO was added in this composition for two reasons: (i) Zn, in small quantities is generally harmless to human body and is a key element for bone development [16] and (ii) Zn can be incorporated in calcium silicates and calcium aluminates lattices increasing in this way the grindability of MTA cement [14].

In this study the MTA cement was obtained by a modified sol-gel method proposed by Voicu *et al.* [12,13,14]. As raw materials tetraethyl orthosilicate (C_6_H_16_O_3_Si—TEOS), aluminium butoxide (C_12_H_27_O_3_Al), zinc acetate (Zn(CH_3_COO)_2∙_2H_2_O) and calcium nitrate (CaNO_3∙_4H_2_O) were used. The main steps of the MTA synthesis were:

(a) aluminum butoxide and acetyl acetone (1:1 molar ratio) were magnetically stirred for 2 h;
(b) calcium nitrate was dissolved in distilled water and magnetically stirred until a clear solution was obtained; then zinc acetate was added and the solution was magnetically stirred for 2 h at 80 °C; next, TEOS was added in this clear solution and the mixture was homogenized until a clear solution was obtained (molar ratio CaO:ZnO:SiO_2_:H_2_O was 1.24:0.04:0.36:10);
(c) the two solutions were mixed for 3 h at room temperature and then kept at 80 °C until a gel was formed.

This gel was maturated for 24 h and then dried at 125 °C for 24 h when a resin type precursor was obtained. The resin-type precursor was thermally treated in a platinum crucible at two different temperatures: 1400 °C/2 h (MTA1) and 1450 °C/3 h (MTA2), respectively. The heating was performed with 10 °C /min and the cooling was performed rapidly in air.

The MTA cements were obtained by the grinding of these clinkers (MTA1 and MTA2) for 15 min in a planetary ball mill (v_rot_ = 150 rot/min). The resulted powder was analyzed by laser granulometry (by means of a Mastersizer 2000 laser granulometer, Malvern, U.K).

The binding properties of MTA cements were assessed on pastes prepared with distilled water (cement to water weight ratio of 3:1). For the assessment of the setting time, 50 cm^3^ of MTA cement paste was filled in a metallic ring (φ = 10 mm, h = 5 mm) and kept on a glass plate in water bath (37 °C and R.H. 80%). The setting time was assessed with a Vicat apparatus) and represents the time elapsed from the moment when the cement powder was mixed with distilled water until the Vicat needle do not leave any imprint on the surface of the paste.

The compressive strength was assessed on paste specimens (cylinders φ = h = 10 mm) cured the first 24 h in the mold, placed on a glass plate in the water bath (37 °C and R.H. 80%); after that the paste specimens were demoulded and stored in distilled water at 37 °C up to 7 or 28 days. The compressive strength was assessed on a Cyber-Tronic testing machine (MATEST, Treviolo, Italy).

The hydration and hardening processes of the MTA cements were assessed on paste specimens prepared and cured as presented above. After 1, 7 and 28 days, the pastes were ground until a fine powder was obtained. This powder was washed with ethyl alcohol and dried at 50 °C for 24 h. The dried powder was analyzed by X-ray diffraction (XRD) and thermogravimentry (TG-DTG) coupled with differential thermal analysis (DTA).

X-ray diffraction analysis was performed using an Empyrean diffractometer (Panalytical, Almelo, Netherland) with CuKα radiation (λ = 1.5418 Å), with scan step of 0.02° and counting time of 0.6 s/step; the diffractometer performs also Rietveld refinement of XRD patterns.

The termogravimetry and differential thermal analysis were performed using a DTG-TA-60 derivatograph (Shimadzu, Kyoto, Japan); the analysis were performed in the 20–1000 °C temperature range, with a heating rate of 10 °C/min, in air.

The microstructure of MTA cement pastes hydrated for 1, 7 and 28 days, was assessed by scanning electronic microcopy (SEM) using a S2600N instgrument (HITACHI, Kyoto, Japan). The specimens for SEM analysis were covered with a thin silver layer deposited by dc-sputtering.

The *in vitro* bioactivity of MTA cements was assessed on cement pastes hydrated for 7 days and soaked in simulated body fluid (SBF) (the specimen area to SBF volume ratio was 0.1 cm^−1^). The paste specimens were stored for 14 days in water bath at 37 °C and then removed, gently rinsed with distilled water in order to remove all the soluble salts and dried at 60 °C for 24 h. The specimen’s surface was then analyzed by XRD, Fourier Transformed Infrared Spectroscopy (FT-IR) and SEM.

FT-IR measurements were performed using a Nicolet™ iS™50 spectrometer (Thermo Scientific, USA) equipped with an ATR module based on diamond crystal. The spectra were recorded over the wavenumber range of 150–1800 cm^−1^ with a resolution of 2 cm^−1^.

Also, in vitro biocompatibility tests were MTT assay and fluorescent microscopy for tracing of living cells:

a) MTT assay

The human endothelial cells line (EAhy923, American Type Culture Collection-ATCC, Manassas, VA, USA) was used to evaluate the biocompatibility of MTA. The cells were cultured in DMEM medium (Sigma-Aldrich, St. Louis, MO, USA) supplemented with 10% fetal bovine serum, 1% penicillin and 1% streptomycin antibiotics (Sigma-Aldrich). To maintain optimal culture conditions, medium was changed twice a week. The biocompatibility was assessed using a MTT assay (CellTiter 96^®^ Non-Radioactive Cell Proliferation Assay, Promega, Fitchburg, WI, USA). This assay is a colorimetric method that allows quantitative assessment of proliferation, cell viability and cytotoxicity. The method is based on the reduction of yellow tetrazolium salt MTT (3-(4,5-dimetylthiazolyl)-2,5-diphenyltetrazolium bromide) to a dark blue formazan by the mitochondrial enzymes. Briefly, the human endothelial cells were grown in 96-well plates, with a seeding density of 3000 cells/well in the presence of MTA for 24–48 h. Then 15 mL Solution I was added and incubated at 37 °C for 4 h. After that the Solution II was added and pipette vigorously to solubilise the formazan crystals. After 1 h the absorbance was read at 570 nm using a spectrophotometer (TECAN, Männedorf, Switzerland).

b) Fluorescent microscopy for tracing of living cells

A second method was additionally used for evaluation of the biocompatibility of MTA based on fluorescent microscopy using the RED CMTPX fluorophore (Life Technologies, Invitrogen, MA, USA, a cell tracker for long-term monitoring of living cells. The CMTPX was added after 7–28 days of cell culture in the presence of MTA for evaluating the viability and morphology of the endothelial. The CMTPX fluorophore was added in the culture medium at a final concentration of 5 μM, incubated for 30 min in order to allow the dye penetration into the cells. Next, the endothelial cells were washed with PBS and visualized by fluorescent microscopy. The photomicrographs were taken with a digital camera driven by Axio-Vision 4.6 software (Carl Zeiss, Oberkochen, Germany).

The control cells were endothelial cells cultivated in the same medium, but without the MTA1 and MTA2 cements.

## 4. Conclusions

The results presented in this paper show that MTA cements with short setting times can be obtained by a sol-gel synthesis route and an adequate thermal treatment. For the MTA cement obtained at a higher temperature e.g., 1450 °C/3 h, the setting time was 15 min, an acceptable value for an endodontic bio-cement.

The experimental results obtained by different analysis techniques (X ray diffraction, thermal analysis, scanning electronic microscopy and FT-IR spectroscopy) showed the presence of a high amount of hydrates (calcium silicate hydrates and calcium aluminate hydrates) in the hardened MTA cement pastes; the good compressive strengths assessed on hardened MTA cement pastes can be correlated with this high amount of hydrates.

The *in vitro* bioassays results demonstrate a high cell viability and good biocompatibility of MTA cements synthesized in this study.

## Figures and Tables

**Table 1:** Granulometric characteristics of MTA1 and MTA2 cements.

Cement	Average Particle Size (μm)	d0.1 (μm) *	d0.9 (μm) **
MTA1	15.03	2.867	31.143
MTA2	10.77	0.690	26.652

* The diameter where 10% of the population lies below this value; ** the diameter where 90% of the population lies below this value.

**Table 2:** Amount of main crystalline compounds present in MTA cements assessed by the Rietveld refinement technique.

Mineralogic Compounds	Specimen	
	MTA1	MTA2
C_3_S (%)	68.60	71.40
C_2_S (%)	13.70	11.80
C_3_A (%)	13.40	15.70
CaO (%)	4.30	1.10

**Table 3:** Setting time and compressive strengths of MTA cements.

Cement	Setting Time (min)	Compressive Strength after 7 Days (MPa)	Compressive Strength after 28 Days (MPa)
MTA 1	55	9.2	18.2
MTA 2	15	12.7	22.9
